# Inhibition of light-induced stomatal opening by allyl isothiocyanate does not require guard cell cytosolic Ca^2+^ signaling

**DOI:** 10.1093/jxb/eraa073

**Published:** 2020-02-27

**Authors:** Wenxiu Ye, Eigo Ando, Mohammad Saidur Rhaman, Md Tahjib-Ul-Arif, Eiji Okuma, Yoshimasa Nakamura, Toshinori Kinoshita, Yoshiyuki Murata

**Affiliations:** 1 School of Agriculture and Biology, Shanghai Jiao Tong University, Shanghai, China; 2 Graduate School of Environmental and Life Science, Okayama University, Tsushima-Naka, Okayama, Japan; 3 Institute of Transformative Bio-Molecule, Nagoya University, Chikusa, Nagoya, Japan; 4 Graduate School of Science, Nagoya University, Chikusa, Nagoya, Japan; 5 Lancaster University, UK

**Keywords:** Allyl isothiocyanate, Arabidopsis, calcium channel, potassium channel, proton pump, stomatal closure, stomatal opening

## Abstract

The glucosinolate–myrosinase system is a well-known defense system that has been shown to induce stomatal closure in Brassicales. Isothiocyanates are highly reactive hydrolysates of glucosinolates, and an isothiocyanate, allyl isothiocyanate (AITC), induces stomatal closure accompanied by elevation of free cytosolic Ca^2+^ concentration ([Ca^2+^]_cyt_) in Arabidopsis. It remains unknown whether AITC inhibits light-induced stomatal opening. This study investigated the role of Ca^2+^ in AITC-induced stomatal closure and inhibition of light-induced stomatal opening. AITC induced stomatal closure and inhibited light-induced stomatal opening in a dose-dependent manner. A Ca^2+^ channel inhibitor, La^3+^, a Ca^2+^chelator, EGTA, and an inhibitor of Ca^2+^ release from internal stores, nicotinamide, inhibited AITC-induced [Ca^2+^]_cyt_ elevation and stomatal closure, but did not affect inhibition of light-induced stomatal opening. AITC activated non-selective Ca^2+^-permeable cation channels and inhibited inward-rectifying K^+^ (K^+^_in_) channels in a Ca^2+^-independent manner. AITC also inhibited stomatal opening induced by fusicoccin, a plasma membrane H^+^-ATPase activator, but had no significant effect on fusicoccin-induced phosphorylation of the penultimate threonine of H^+^-ATPase. Taken together, these results suggest that AITC induces Ca^2+^ influx and Ca^2+^ release to elevate [Ca^2+^]_cyt_, which is essential for AITC-induced stomatal closure but not for inhibition of K^+^_in_ channels and light-induced stomatal opening.

## Teaser

Allyl isothiocyanate (AITC) induces Ca^2+^ influx and Ca^2+^ release to elevate [Ca^2+^]_cyt_, which is essential for AITC-induced stomatal closure but not for inhibition of K^+^_in_ channels or light-induced stomatal opening.

## Introduction

Stomata, surrounded by pairs of guard cells, function as the main window for gas exchange and are in the frontline for defense against microbe invasion in the phyllosphere. To deal with the changing growth and environmental cues, guard cells have evolved to be specialist responders to various stimuli, such as light, drought stress, CO_2_, phytohormones, and microbe-derived signals, resulting in stomatal movement (Murata *et al.*, 2015; Ye & Murata, 2016; Inoue & Kinoshita, 2017; Takahashi *et al.*, 2018; Zhang *et al.*, 2018).

The glucosinolate–myrosinase system is a well-known defense system against herbivores and pathogens in Brassicales (Halkier & Gershenzon, 2006; Bednarek *et al.*, 2009; Clay *et al.*, 2009). Myrosinases are highly abundant proteins in guard cells and required for abscisic acid (ABA)-induced stomatal movement (Zhao *et al.*, 2008; Islam *et al.*, 2009). Recent results suggest that aliphatic glucosinolates are involved in stomatal movement regulated by ABA and auxin (Zhu *et al.*, 2014; Salehin *et al.*, 2019). Isothiocyanates (ITCs) are highly reactive hydrolysates of glucosinolates and an ITC, allyl isothiocyanate (AITC), induces stomatal closure in Arabidopsis (Khokon *et al.*, 2011; Hossain *et al.*, 2014). AITC reversibly induces stomatal closure in *Vicia faba*, which does not belong to Brassicales (Sobahan *et al.*, 2015). Since many ITCs including AITC are volatile, the results imply that ITCs function as signals for plant–plant interaction (Sobahan *et al.*, 2015). To elucidate the mechanism of AITC-induced stomatal closure, it has been shown that AITC induces production of reactive oxygen species (ROS) and elevation of free cytosolic Ca^2+^ concentration ([Ca^2+^]_cyt_) in guard cells (Khokon *et al.*, 2011). Pharmacological studies indicate that ROS are essential for AITC-induced stomatal closure (Hossain *et al.*, 2014; Sobahan *et al.*, 2015).

ABA-induced stomatal movement has long been studied as a signaling model in guard cells. ABA induces stomatal closure and inhibits light-induced stomatal opening. It was widely thought that the molecular mechanisms underlying these two responses overlap, but recent studies provide evidence that the two signaling pathways can be genetically dissected (Yin *et al.*, 2013). It is important to find out whether other guard cell signaling pathways can be dissected. Though AITC induces stomatal closure, it remains unknown whether it inhibits light-induced stomatal opening.

Cytosolic Ca^2+^ is a well-known second messenger in both stomatal closure and stomatal opening (Shimazaki *et al.*, 2007; Hubbard *et al.*, 2012; Murata *et al.*, 2015). Exogenous Ca^2+^ application is known to induce stomatal closure and inhibit light-induced stomatal opening (Peiter *et al.*, 2005; Islam *et al.*, 2010). Elevations of [Ca^2+^]_cyt_ are triggered by influx of Ca^2+^ from the apoplast and release of Ca^2+^ from intracellular stores in guard cell signaling (Leckie *et al.*, 1998; Hamilton *et al.*, 2000; Pei *et al.*, 2000; Lemtiri-Chlieh *et al.*, 2003; Garcia-Mata *et al.*, 2003). The influx of Ca^2+^ is carried by non-selective Ca^2+^-permeable cation channels (I_Ca_ channels) that are activated by plasma membrane hyperpolarization (Hamilton *et al.*, 2000; Pei *et al.*, 2000). Pharmacological studies using nicotinamide (NA), an inhibitor of cyclic adenosine diphosphate ribose (cADPR) synthesis (Dodd *et al.*, 2007), suggest that the release of Ca^2+^ is mediated by a cADPR-dependent pathway (Allen *et al.*, 1995; Klüsener *et al.*, 2002; Hossain *et al.*, 2014). It is known that cADPR stimulates ryanodine receptors in the endoplasmic reticulum in animal cells, but homologous genes have not been identified in the Arabidopsis genome (Hetherington and Brownlee, 2004), and therefore, it is likely that cADPR functions via a different mechanism in plant cells. Elevation of [Ca^2+^]_cyt_ activates S-type anion channels in guard cells and inhibits plasma membrane H^+^-ATPase and inward-rectifying potassium (K^+^_in_) channels (Schroeder & Hagiwara, 1989; Kinoshita *et al.*, 1995; Siegel *et al.*, 2009; Wang *et al., 2013*a**). On the other hand, these transporters are subject to regulation by other components in addition to Ca^2+^, which probably determine signaling specificity (Allen *et al.*, 2002; Siegel *et al.*, 2009; Xue *et al.*, 2011; Ye *et al.*, 2015). Furthermore, a Ca^2+^-independent pathway exists to regulate stomatal movement (Roelfsema & Hedrich, 2010; Laanemets *et al.*, 2013). Though AITC induces [Ca^2+^]_cyt_ elevation, it remains unknown how AITC does this and whether the elevation is essential for AITC-induced stomatal movement. The effects of AITC on I_Ca_ channels and K^+^_in_ channels in guard cells remain to be clarified.

In the present study, we aimed to investigate the role of [Ca^2+^]_cyt_ elevation in AITC-induced stomatal closure and inhibition of light-induced stomatal opening in Arabidopsis. The presented results reveal that AITC induced influx of Ca^2+^ through activation of I_Ca_ channels and Ca^2+^ release possibly through a cADPR-dependent pathway, resulting in the elevation of [Ca^2+^]_cyt_ in guard cells. While [Ca^2+^]_cyt_ elevation was essential for AITC-induced stomatal closure, it was not essential for inhibition of light-induced stomatal opening. The inhibition was attributed to Ca^2+^-independent inhibition of K^+^_in_ channels by AITC in guard cells.

## Materials and methods

### Plant materials and growth conditions

Arabidopsis wild-type plants (Columbia) were grown in pots containing a mixture of 70% (v/v) vermiculite (Asahi-kogyo, Okayama, Japan) and 30% (v/v) Kureha soil (Kureha Chemical, Tokyo, Japan) in a growth chamber (photon flux density of 80 µmol m^−2^ s^−1^ under a 16 h light–8 h dark regime). The temperature and relative humidity in the growth chamber were 22±2 °C and 60±10%, respectively. Twice or three times a week, 0.1% Hyponex solution (Hyponex, Osaka, Japan) as a fertilizer was provided to the plants. All the plants used for experiments were from 4 to 6 weeks old.

### Stomatal aperture measurement

Fully expanded young leaves from 4- to 5-week-old plants were excised for stomatal aperture measurements as described previously (Ye *et al.*, 2013). For assays of light-induced stomatal opening, leaves were floated on assay solution containing 5 mM KCl, 50 μM CaCl_2_, and 10 mM MES–Tris (pH 6.15) with their adaxial surface upward in the dark for 2 h to close the stomata. After adding AITC, the leaves were kept in the light (80 μmol m^−2^ s^−1^) for 2 h before measurement. For assays of stomatal closure, leaves were floated on the assay solution in the light for 2 h to open the stomata. Then AITC was added, and the leaves were kept in the light for 2 h before measurement. Inhibitors were added 30 min before AITC treatment. For measurement of stomatal apertures, the leaves were shredded for 30 s, and epidermal tissues were collected using nylon mesh. Thirty stomatal apertures were measured for each sample.

### Imaging of [Ca^2+^]_cyt_ in guard cells

Four- to six-week-old wild-type plants expressing YC3.6 were used for the measurement of [Ca^2+^]_cyt_ in guard cells as described previously (Ye et al., 2013, 2015). The abaxial side of an excised leaf was gently mounted on a glass slide with a medical adhesive (stock no. 7730; Hollister) followed by removal of the adaxial epidermis and the mesophyll tissue with a razor blade in order to keep the lower epidermis intact on the slide. The remaining abaxial epidermis was incubated in solution containing 5 mM KCl, 50 μM CaCl_2_, and 10 mM MES–Tris (pH 6.15) in the light for 2 h at 22 °C to promote stomatal opening. Turgid guard cells were used to measure [Ca^2+^]_cyt_. The observation chamber was perfused with the solution using a peristaltic pump. Guard cells were incubated in the absence of AITC for 5 min and then in the presence of AITC. Inhibitors were added at the time point indicated. For dual-emission ratio imaging of YC3.6, we used an FF-02-438/24-25 (Semrock) excitation filter, a 445DRLP (Omega) dichroic mirror, an XF3075 480AF30 (Omega) emission filter for cyan fluorescent protein (CFP), and an XF3011 535DF25 (Omega) emission filter for yellow fluorescent protein (YFP). The CFP and YFP fluorescence intensity of guard cells were imaged and analysed using the W-View system and AQUA COSMOS software (Hamamatsu Photonics). CFP and YFP fluorescence were simultaneously monitored.

### Patch-clamp measurement

Current measurements of I_Ca_ and K^+^_in_ channels in Arabidopsis guard cells were performed as described previously (Mori *et al.*, 2006; Ye *et al.*, 2013). Arabidopsis guard cell protoplasts were prepared from rosette leaves. For I_Ca_ current measurement, the pipette solution contained 10 mM BaCl_2_, 0.1 mM dithiothreitol, 4 mM EGTA, and 10 mM HEPES–Tris, pH 7.1. The bath solution contained 100 mM BaCl_2_, 0.1 mM dithiothreitol, and 10 mM MES–Tris, pH 5.6. For K^+^_in_ channel current measurement, the pipette solution contained 30 mM KCl, 70 mM K-Glu, 2 mM MgCl_2_, 2.4 mM CaCl_2_ (free Ca^2+^ concentration, 150 nM), 6.7 mM EGTA, and 10 mM HEPES–Tris, pH 7.1. The bath solution contained 30 mM KCl, 2 mM MgCl_2_, 40 mM CaCl_2_, and 10 mM MES–Tris, pH 5.5. In all cases, osmolality was adjusted to 500 mmol kg^−1^ (pipette solutions) and 485 mmol kg^−1^ (bath solutions) with D-sorbitol.

### KAT1 current recording in *Xenopus laevis* oocytes

The expression of KAT1 in *Xenopus laevis* oocytes and current recording were performed according to our previous method (Islam *et al.*, 2015). Before recording, the microinjected oocytes were incubated in ND96 solution containing 94 mM NaCl, 2 mM KCl, 1 mM CaCl_2_, 1 mM MgCl_2_, and 5 mM HEPES (pH 7.5) with different concentrations of AITC for 2 h.

### Immunohistochemical detection of the plasma membrane H^+^-ATPase in guard cells using a whole leaf

Immunohistochemistry using a whole leaf was performed according to Sauer *et al.* (2006) and Hayashi *et al.* (2011) with modifications. Mature leaves were harvested from dark-adapted plants and floated on the basal buffer (5 mM MES–BTP (pH 6.5), 50 mM KCl, and 0.1 mM CaCl_2_) containing 50 µM AITC for 20 min in the dark. After AITC treatment, 10 µM fusicoccin was added to the buffer and kept for a further 10 min. For the control, 0.1% (v/v) dimethyl sulfoxide was added to the buffer. After treatment, leaves were put into a syringe with fixative (4% (w/v) formaldehyde freshly prepared from paraformaldehyde and 0.3% (v/v) glutaraldehyde in 50 mM PIPES–NaOH (pH 7.0), 5 mM MgSO_4_, and 5 mM EGTA), and negative pressure applied several times to infiltrate the fixative, followed by immersion in the solution for 1 h in the dark at room temperature. After washing with phosphate-buffered saline (PBS; 137 mM NaCl, 8.1 mM Na_2_HPO_4_, 2.68 mM KCl, and 1.47 mM KH_2_PO_4_), chlorophyll was removed by pure methanol (20 min incubation at 37 °C three or four times). Then, central areas of the leaves were cut out, and incubated with xylene at 37 °C for 2 min, pure ethanol at room temperature for 5 min, and 50% (v/v; in PBS) ethanol at room temperature for 5 min, and washed with Milli-Q water twice. The material was transferred to MAS-coated microscope slides (Matsunami) with a droplet of water, where the abaxial side of the leaf was attached to the slide, and freeze–thaw treatment applied followed by complete drying overnight at room temperature. Dried samples were rehydrated by PBS for 5 min at room temperature, and digested with 4% (w/v) Cellulase Onozuka R-10 (Yakult) with 0.5% (w/v) Macerozyme R-10 (Yakult) in PBS for 1 h at 37 °C. After digestion, leaf tissue except for the abaxial epidermis attached on the slide was removed stereomicroscopically in PBS, and the left epidermal tissue was washed four times for 5 min each with PBS, then permeabilized with 3% (v/v) IGEPAL CA-630 (MP Biomedicals) with 10% (v/v) dimethyl sulfoxide in PBS for 1 h at room temperature. Samples were washed five times for 5 min each with PBS and incubated with blocking solution (3% (w/v) bovine serum albumin Fraction V (BSA; Thermo Fisher Scientific) in PBS) for 1 h at room temperature. The primary antibody (anti-pThr; Hayashi *et al.*, 2010) was treated with a dilution of 1:1000 in the blocking solution at 4 °C overnight. Samples were washed five times for 5 min each with PBS; secondary antibody (Alexa Fluor 488-conjugated goat anti-rabbit IgG; Thermo Fisher Scientific) with a dilution of 1:500 in the blocking solution was applied at 37 °C for 3 h, followed by washing five times for 5 min each with PBS. The specimens were covered by a coverglass with 50% (v/v) glycerol. Fluorescence images were obtained (Hayashi *et al.*, 2011) and analysed by ImageJ software (National Institutes of Health).

### Statistical analysis

The significance of differences between datasets was assessed by Student’s *t*-test and analysis of variance (ANOVA) with Tukey’s test. The response of [Ca^2+^]_cyt_ was assessed by χ ^2^ test. Differences were considered significant for *P*<0.05.

## Results

### Effect of inhibitors of Ca^2+^ influx and Ca^2+^ release pathways on allyl isothiocyanate-induced stomatal movement

**Fig. 1.**
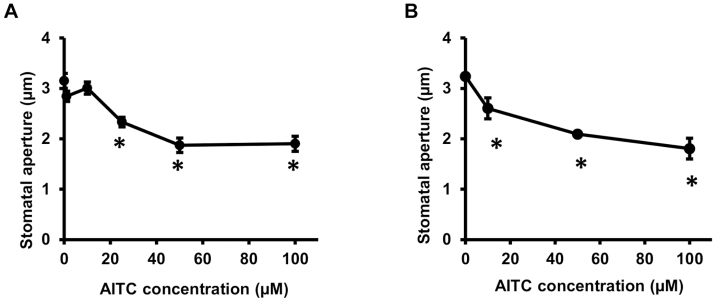
Effect of AITC on stomatal movement. (A) Stomatal closure induced by AITC. (B) Inhibition by AITC of light-induced stomatal opening. Averages from three independent experiments (90 stomata in total per bar) are shown. Error bars represent standard error of the mean (*n*=3). *Statistical significance compared with 0 μM AITC (*P*<0.05).

To investigate AITC-induced stomatal closure, intact leaves were initially incubated in the light to open the stomata followed by AITC treatment in the light. For investigation of AITC inhibition of light-induced stomatal opening, leaves were initially incubated in the dark to close the stomata followed by AITC treatment in the light. AITC induced stomatal closure in a dose-dependent manner (Fig. 1A), which is consistent with previous results (Khokon *et al.*, 2011). In addition, AITC inhibited light-induced stomatal opening in a dose-dependent manner (Fig. 1B).

**Fig. 2.**
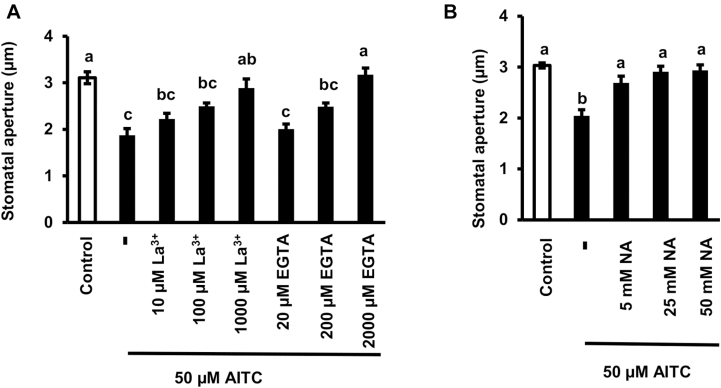
Effects of La^3+^, EGTA, and NA on AITC-induced stomatal closure. (A) Effects of La^3+^ and EGTA on AITC-induced stomatal closure. (B) Effect of NA on AITC-induced stomatal closure. Averages from three independent experiments (90 stomata in total per bar) are shown. Error bars represent standard error of the mean (*n*=3). Different letters indicate statistical significance between groups (*P*<0.05).

Previous results have shown that AITC induces stomatal closure accompanied by [Ca^2+^]_cyt_ elevation. However, it remains unknown whether the [Ca^2+^]_cyt_ elevation is essential for AITC-induced stomatal closure. Application of a Ca^2+^ channel blocker, La^3+^, and a Ca^2+^ chelator, EGTA, inhibited AITC-induced stomatal closure in a dose-dependent manner (Fig. 2A). Nicotinamide, an inhibitor of Ca^2+^ release, also inhibited AITC-induced stomatal closure in a dose-dependent manner (Fig. 2B). These results suggest that [Ca^2+^]_cyt_ elevation induced by Ca^2+^ influx and Ca^2+^ release is essential for AITC-induced stomatal closure.

**Fig. 3.**
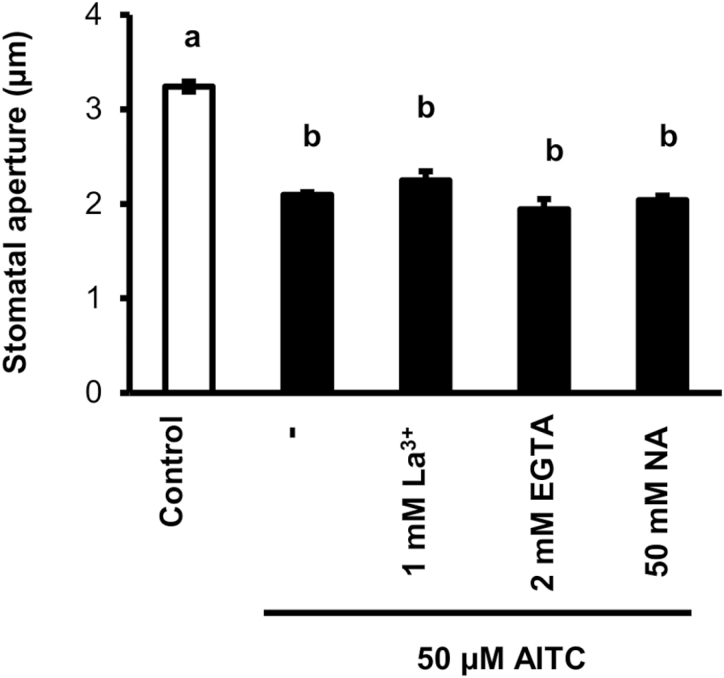
Effects of La^3+^, EGTA, and NA on inhibition by AITC of light-induced stomatal opening. Averages from three independent experiments (90 stomata in total per bar) are shown. Error bars represent standard error of the mean (*n*=3). Different letters indicate statistical significance between groups (*P*<0.05).

On the other hand, the three inhibitors even at the highest concentration for stomatal closure assay (Fig. 2) did not significantly affect AITC inhibition of light-induced stomatal opening (Fig. 3). These results suggest that [Ca^2+^]_cyt_ elevation is not essential for AITC inhibition of light-induced stomatal opening.

Since intact leaves were used for stomatal movement assay, AITC and inhibitors have to be transported via the petiole and/or penetrate cuticle directly to reach the guard cells. Therefore, it is possible that concentrations of AITC and inhibitors in the apoplast of intact leaves are lower than those in the assay solution.

### Effect of inhibitors of Ca^2+^ influx and Ca^2+^ release pathways on allyl isothiocyanate-induced [Ca^2+^]_cyt_ elevation

**Fig. 4.**
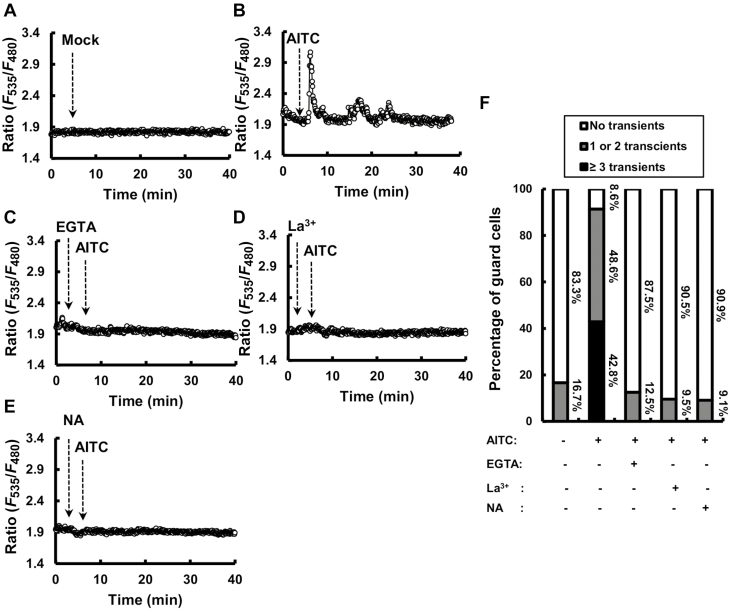
Effects of La^3+^, EGTA, and NA on AITC-induced elevation of [Ca^2+^]_cyt_ in guard cells. (A–E) Representative traces of fluorescence emission ratios (535/480 nm) showing transient [Ca^2+^]_cyt_ elevations in guard cells; 1 mM La^3+^, 2 mM EGTA, and 50 mM NA were added 4 min before 50 µM AITC treatment. (F) Percentage of guard cells showing 0, 1 or 2, or ≥3 transient [Ca^2+^]_cyt_ elevations. [Ca^2+^]_cyt_ elevations were counted when changes in fluorescence emission ratios were ≥0.1 U from the baseline.

Previous results have shown that AITC induced [Ca^2+^]_cyt_ elevations in guard cells (Khokon *et al.*, 2011). Here, we investigated how AITC induces [Ca^2+^]_cyt_ elevations in guard cells from isolated epidermal tissues. Mock treatment showed [Ca^2+^]_cyt_ elevations in 16.7% of guard cells (Fig. 4A, F), whereas application of 50 μM AITC induced elevations in 91.4% of wild-type guard cells (Fig. 4B, F), which is consistent with previous results (Khokon *et al.*, 2011). Application of 1 mM La^3+^, 2 mM EGTA and 50 mM NA abolished the AITC-induced [Ca^2+^]_cyt_ elevations (Fig. 4C–F). These results indicate that the inhibitors do suppress AITC-induced [Ca^2+^]_cyt_ elevations and that both Ca^2+^ influx and Ca^2+^ release contribute to the [Ca^2+^]_cyt_ elevations.

Other Ca^2+^ channel inhibitors, Gd^3+^, verapamil (VERA) and ruthenium red (RR) (Allen *et al.*, 1995; McAinsh *et al.*, 1995; Cessna *et al.*, 1998), were used to further investigate AITC-induced [Ca^2+^]_cyt_ elevations. One millimolar Gd^3+^ abolished and 1 mM VERA slightly impaired [Ca^2+^]_cyt_ elevations induced by 50 μM AITC (Fig. S1A, B, D). On the other hand, RR at 100 μM did not affect the [Ca^2+^]_cyt_ elevations significantly (Fig. S1C, D). These results suggest that Gd^3+^- and VERA-sensitive Ca^2+^ channels are involved in AITC-induced [Ca^2+^]_cyt_ elevations.

### Effect of allyl isothiocyanate on I_Ca_ currents and K^+^_in_ currents in guard cell protoplasts

**Fig. 5.**
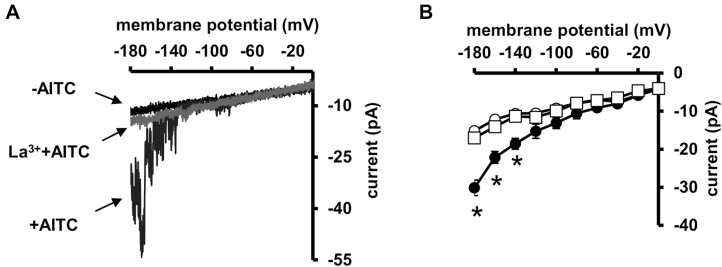
Activation of I_Ca_ channel currents by AITC in guard cell protoplasts (GCPs). (A) Representative I_Ca_ current traces in GCPs. (B) Average of current–voltage curves for AITC activation of I_Ca_ currents in GCPs (*n*=5) as recorded in (A) (open circles, control; filled circles, 50 μM AITC; open square, La^3+^+AITC). A ramp voltage protocol from +20 to −180 mV (holding potential, 0 mV; ramp speed, 200 mV s^−1^) was used. After attaining the whole-cell configuration, GCPs were recorded to obtain control data. To obtain data for ATIC treatment and La^3+^+AITC treatment, recordings were performed sequentially after extracellularly adding AITC and 1 mM La^3+^. The GCPs were measured 16 times to get averages for each recording. The interpulse period was 1 min. *Statistical significance (*P*<0.05). Results are from five independent experiments; error bars indicate SE.

Application of 50 μM AITC significantly activated I_Ca_ currents when the membrane was hyperpolarized, and the currents were inhibited by the Ca^2+^ channel inhibitor La^3+^ (Fig. 5). These results indicate that AITC induces Ca^2+^ influx through activation of I_Ca_ channels.

**Fig. 6.**
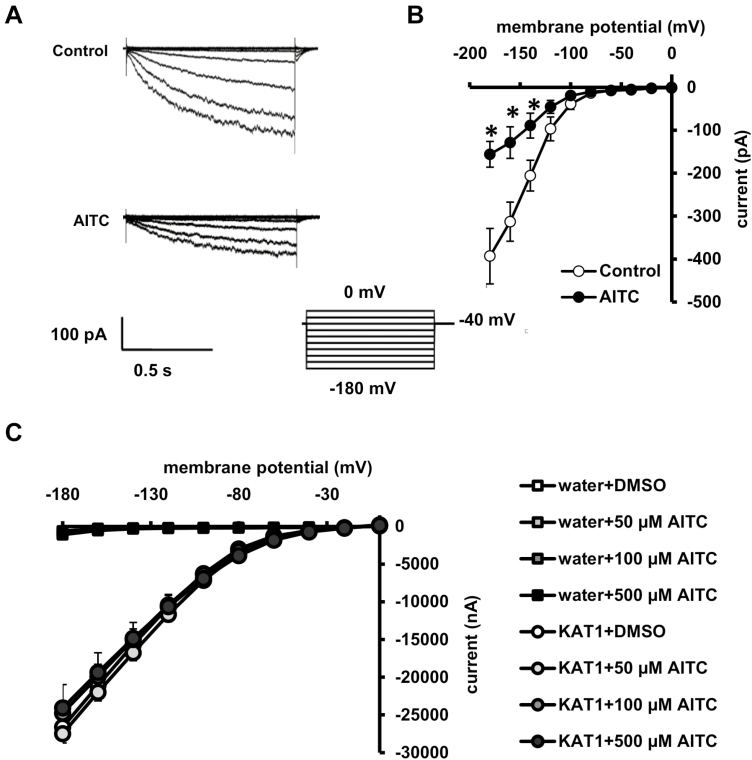
Effect of AITC on K^+^_in_ currents in GCPs and KAT1 activity expressed in *Xenopus* oocytes. (A) K^+^_in_ currents in GCPs treated without (top trace) or with (bottom trace) 50 μM AITC. (B) Steady-state current–voltage relationship for AITC inhibition of K^+^_in_ currents in WT GCPs as recorded in (A) (open circles, control; filled circles, AITC). The voltage protocol was stepped up from 0 mV to −180 mV in 20 mV decrements (holding potential, −40 mV). GCPs were treated with AITC for 2 h before recordings. Each data point was obtained from at least seven GCPs in more than five independent experiments. Error bars represent standard errors. *Statistical significance compared with Control (*P*<0.05). (C) Steady-state current–voltage relationship for KAT1-mediated currents in *Xenopus* oocytes. Oocytes were treated with AITC for 2 h before recordings. The voltage protocol was stepped up from 0 mV to −180 mV in 20 mV decrements (holding potential, −40 mV) with a pulse duration of 3 s. Each data point was obtained from seven oocytes in more than three independent experiments. Error bars represent standard errors.

Potassium influx through K^+^_in_ channels is critical for stomatal opening (Kwak *et al.*, 2001; Takahashi *et al.*, 2013). Resting [Ca^2+^]_cyt_ has been shown to be in the range of 120–150 nM in guard cells (Grabov & Blatt, 1999; Siegel *et al.*, 2009). Since AITC inhibition of light-induced stomatal opening is not dependent on [Ca^2+^]_cyt_ elevation, the effect of AITC on K^+^_in_ currents at resting [Ca^2+^]_cyt_ buffered to 150 nM was investigated. Application of 50 µM AITC significantly suppressed K^+^_in_ currents in guard cell protoplasts (Fig. 6A, B), indicating that AITC suppresses K^+^_in_ channels in a Ca^2+^-independent manner.

The effect of AITC on a major K^+^_in_ channel in guard cells, KAT1, was investigated using the two-electrode voltage-clamp technique. AITC at 50, 100, and 500 µM had no significant effect on the currents seen in *Xenopus* oocytes expressing KAT1 (Fig. 6C).

### Effect of allyl isothiocyanate on fusicoccin-induced stomatal opening and phosphorylation of penultimate threonine of plasma membrane H^+^-ATPases

**Fig. 7.**
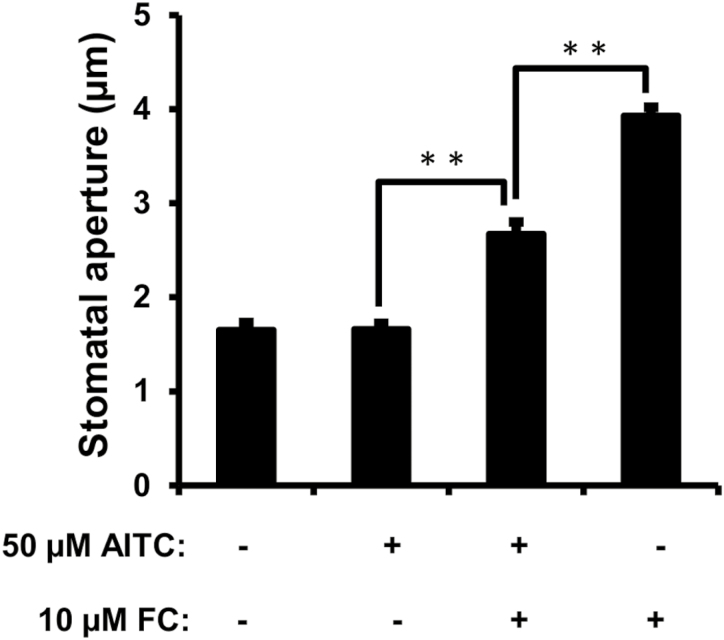
Effect of AITC on FC-induced stomatal opening in the dark. Averages from three independent experiments (90 stomata in total per bar) are shown. AITC was added 30 min before FC treatment. Error bars represent standard error of the mean (*n*=3). *Statistical significance (*P*<0.05).

**Fig. 8.**
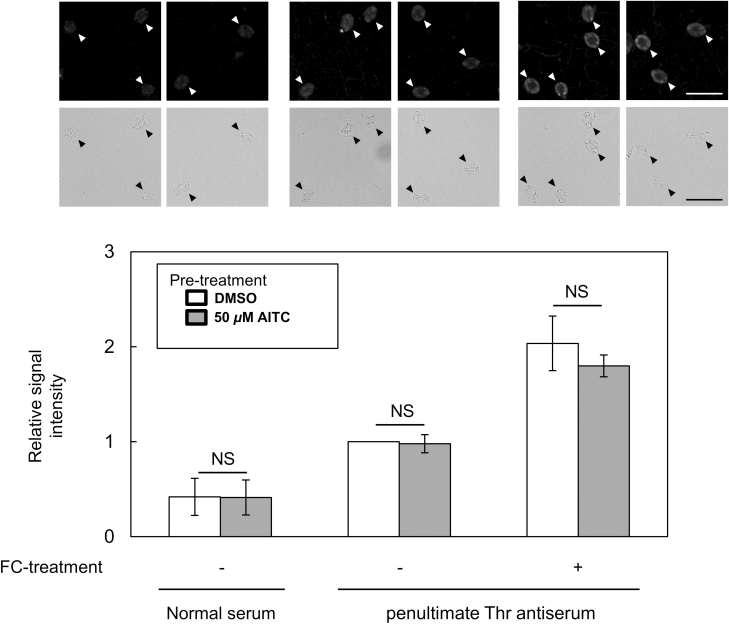
Immunohistochemical detection of FC-induced penultimate Thr phosphorylation of H^+^-ATPases in guard cell plasma membrane. The vertical scale represents fluorescence levels of guard cells detected using penultimate Thr antiserum as primary antibody and Alexa Fluor 488-conjugated goat anti-rabbit IgG as secondary antibody. The fluorescence level was measured by ImageJ, expressed as relative values normalized to that of mock treatment. Typical fluorescence and the corresponding bright field images are shown at the top. Arrowhead indicates the guard cells. Scale bar: 50 µm. Data represent means with SDs (*n*=3). NS, no significant difference observed (*P*>0.1; Student’s *t* test). Results are from more than three independent experiments.

To further investigate how AITC inhibits stomatal opening, the effect of AITC on stomatal opening induced by a plasma membrane H^+^-ATPase activator, fusicoccin (FC), was investigated. Treatment of 50 μM AITC significantly inhibited FC-induced stomatal opening in the dark (Fig. 7). Since FC induces stomatal opening through activation of H^+^-ATPases by increasing the phosphorylation level of the penultimate Thr (penThr) of H^+^-ATPases (Kinoshita & Shimazaki, 2001), the effect of AITC on FC-induced penThr phosphorylation was investigated. Application of 50 μM AITC did not affect FC-induced penThr phosphorylation significantly (Fig. 8). Taken together, these results indicate that AITC inhibits FC-induced stomatal opening without affecting penThr phosphorylation.

## Discussion

### Allyl isothiocyanate induces stomatal movement like many other abiotic and biotic stimuli

The glucosinolate–myrosinase system is widely known to be activated by tissue damage caused by herbivory, and hydrolysis products of glucosinolates have repellent effects on many herbivores (Halkier & Gershenzon, 2006). Studies have also shown that hydrolysis of glucosinolates is induced in live cells by fungal and bacterial infection and plant hormones such as ABA (Zhao *et al.*, 2008; Bednarek *et al.*, 2009; Clay *et al.*, 2009; Fan *et al.*, 2011; Zhu *et al.*, 2014; Andersson *et al.*, 2015). Guard cell responses to ABA were impaired in aliphatic glucosinolate-deficient (Zhu *et al.*, 2014) and myrosinase-deficient (Zhao *et al.*, 2008; Islam *et al.*, 2009) mutants, suggesting that hydrolysis products of glucosinolates function as signaling component in ABA signaling. There is also evidence for involvement of glucosinolate hydrolysis in signaling induced by bacterial flagellin peptide, flg22 (Clay *et al.*, 2009). These results motivate further study of the signaling by the hydrolysis product of glucosinolates. Among the hydrolysis products, isothiocyanates are highly active and their physiological functions in both abiotic and biotic stress are under intensive investigation (Hara *et al.*, 2013; Andersson *et al.*, 2015). Allyl isothiocyanate is biosynthesized in Arabidopsis (Lambrix *et al.*, 2001) and is one of the most studied isothiocyanates so far (Khokon *et al.*, 2011; Hossain *et al.*, 2013; Åsberg *et al.*, 2015; Øverby *et al.*, 2015; Sobahan *et al.*, 2015). While AITC at concentrations above millimolar is detrimental (Hara *et al.*, 2010), AITC at lower concentrations functions as a signal to trigger physiological events (Khokon *et al.*, 2011; Sobahan *et al.*, 2015). It was also suggested that AITC, as a volatile compound, functions as a signal for plant–plant interaction (Sobahan *et al.*, 2015). Previous results have shown that AITC reversibly induces stomatal closure (Khokon *et al.*, 2011; Hossain *et al.*, 2013; Sobahan *et al.*, 2015). In the present study, it was further revealed that AITC also inhibited light-induced stomatal opening (Fig. 1). Many abiotic and biotic stimuli, such as ABA and flg22, also induce both stomatal closure and inhibition of light-induced stomatal opening (Melotto *et al.*, 2006; Zhang *et al.*, 2008; Yin *et al.*, 2013). Therefore, it is likely that there is crosstalk between AITC signaling and signaling induced by other stimuli including ABA and flg22.

### Allyl isothiocyanate induces Ca^2+^ influx and Ca^2+^ release leading to [Ca^2+^]_cyt_ elevations

Activation of I_Ca_ channels in guard cells is triggered by many stimuli, such as ABA, methyl jasmonate (MeJA), yeast elicitor, and amino acids (Murata *et al.*, 2001; Munemasa *et al.*, 2007; Ye *et al.*, 2013; Yoshida *et al.*, 2016; Kong *et al.*, 2016). In the present study, AITC activated I_Ca_ channels in guard cells (Fig. 5). On the other hand, the molecular nature of I_Ca_ channels is mostly unknown. It is known that AITC activates transient receptor potential ankyrin 1, a Ca^2+^ channel, by reacting with several Cys residues in its N-terminus to induce Ca^2+^ influx in neuron cells (Bautista *et al.*, 2006; Hinman *et al.*, 2006). However, plants do not harbor genes related to transient receptor potential channels in animal cells (Hedrich, 2012). Studies have identified that hyperosmolality-induced [Ca^2+^]_cyt_ increase 1 (OSCA1) family members, glutamate receptor homologs, and cyclic nucleotide-gated channels are I_Ca_ channels activated by different stimuli (Wang *et al.*, 2013*b*; Yuan *et al.*, 2014; Kong *et al.*, 2016; Tian *et al.*, 2019). It would be interesting to investigate whether these channels are activated by AITC.

Previous studies have shown not only Ca^2+^ influx but also Ca^2+^ release from intracellular stores is essential for [Ca^2+^]_cyt_ elevations induced by ABA, methyl jasmonate and flg22 (Leckie *et al.*, 1998; Hamilton *et al.*, 2000; Pei *et al.*, 2000; Garcia-Mata *et al.*, 2003; Lemtiri-Chlieh *et al.*, 2003; Munemasa *et al.*, 2007; Hossain *et al.*, 2014; Thor and Peiter, 2014). ABA and MeJA recruit a Ca^2+^ release pathway involving cADPR, which is sensitive to NA (Allen *et al.*, 1995; Klüsener *et al.*, 2002; Hossain *et al.*, 2014). In the present study, NA abolished AITC-induced [Ca^2+^]_cyt_ elevations, suggesting that the NA-sensitive Ca^2+^ release mechanism is conserved among different signaling pathways.

### Elevation of [Ca^2+^]_cyt_ is essential for allyl isothiocyanate-induced stomatal closure but not inhibition of light-induced stomatal opening

The driving force for stomatal closure is membrane depolarization induced by activation of S-type anion channels, while the driving force for stomatal opening is membrane hyperpolarization induced by activation of H^+^-ATPase. Ca^2+^ positively regulates S-type anion channels but negatively regulates H^+^-ATPase through Ca^2+^ sensor-dependent pathways (Schroeder & Hagiwara, 1989; Kinoshita *et al.*, 1995), and thus Ca^2+^ is essential for stomatal closure induced by many stimuli such as ABA, MeJA, and yeast elicitor (Mori *et al.*, 2006; Munemasa *et al.*, 2011; Ye *et al.*, 2013). Recent studies have shown quadruple loss-of-function mutations of ABA receptors impair ABA-induced stomatal closure and [Ca^2+^]_cyt_ elevation but not inhibition of light-induced stomatal opening (Wang *et al.*, 2013*a*; Yin *et al.*, 2013). These results highlight that the signaling pathways underlying stomatal closure and inhibition of stomatal opening can be genetically dissected. In the present study, inhibition of [Ca^2+^]_cyt_ by three inhibitors impaired AITC-induced stomatal closure but not inhibition of light-induced stomatal opening (Figs 2, 3), indicating [Ca^2+^]_cyt_ elevation is essential for AITC-induced stomatal closure but not inhibition of light-induced opening. Taken together, these results suggest that signaling leading to stomatal closure does not overlap with signaling leading to inhibition of light-induced stomatal opening in different stress responses.

Inhibition of K^+^_in_ channels suppresses stomatal opening driven by activation of H^+^-ATPase (Kwak *et al.*, 2001; Takahashi *et al.*, 2013). In the present study, AITC inhibited K^+^_in_ channels in the absence of [Ca^2+^]_cyt_ elevation (Fig. 6). Further results showed that AITC inhibited FC-induced stomatal opening but not phosphorylation of penThr (Figs 7, 8). These results again suggest that inhibition of K^+^_in_ channels contributes to AITC inhibition of light-induced stomatal opening.

As an electrophile, AITC is prone to forming covalent adducts with amino acid residues, such as Cys, Lys, and His, to modify protein function. It is known that the covalent modification of Cys is reversible under physiological conditions (Nakamura and Miyoshi, 2010; Higdon *et al.*, 2012). It has been shown that two electrophiles, acrolein and methylglyoxal, inhibit K^+^_in_ channels in guard cells and target KAT1, a main K^+^_in_ channel in guard cells, expressed in a heterologous system using *Xenopus* oocytes (Hoque *et al.*, 2012; Islam *et al.*, 2015, 2016). However, our experiments show that AITC at concentration of 50, 100, and 500 µM did not significantly affect KAT1 activity expressed in oocytes (Fig. 6C), suggesting that AITC does not directly modify KAT1. It has been reported that one AITC target is microtubules in Arabidopsis (Øverby *et al.*, 2015) and microtubules are critical for light-induced stomatal opening (Marcus *et al.*, 2001; Eisinger *et al.*, 2012). In the future, it would be interesting to investigate whether AITC modulates K^+^_in_ channels through a microtubule-dependent pathway.

## Conclusion

The presented results suggest that AITC triggers Ca^2+^ influx and Ca^2+^ release to induce [Ca^2+^]_cyt_ elevation, which is essential for AITC-induced stomatal closure but not for inhibition of K^+^_in_ channels or light-induced stomatal opening.

## Supplementary data

Supplementary data are available at *JXB* online.

Fig. S1. Effects of Gd^3+^, VERA, and RR on AITC-induced elevation of [Ca^2+^]_cyt_ in guard cells.

eraa073_suppl_Supplementary_Figure_S1
