# Supplementary material for: Inhibition of light-induced stomatal opening by allyl isothiocyanate does not require guard cell cytosolic Ca2+ signaling
Source: J Exp Bot. 2020 Feb 27;71(10):2922–32. doi: 10.1093/jxb/eraa073 (PMC7260714; doi:10.1093/jxb/eraa073)
Supplement: eraa073_suppl_Supplementary_Figure_S1 [file eraa073_suppl_supplementary_figure_s1.pdf]

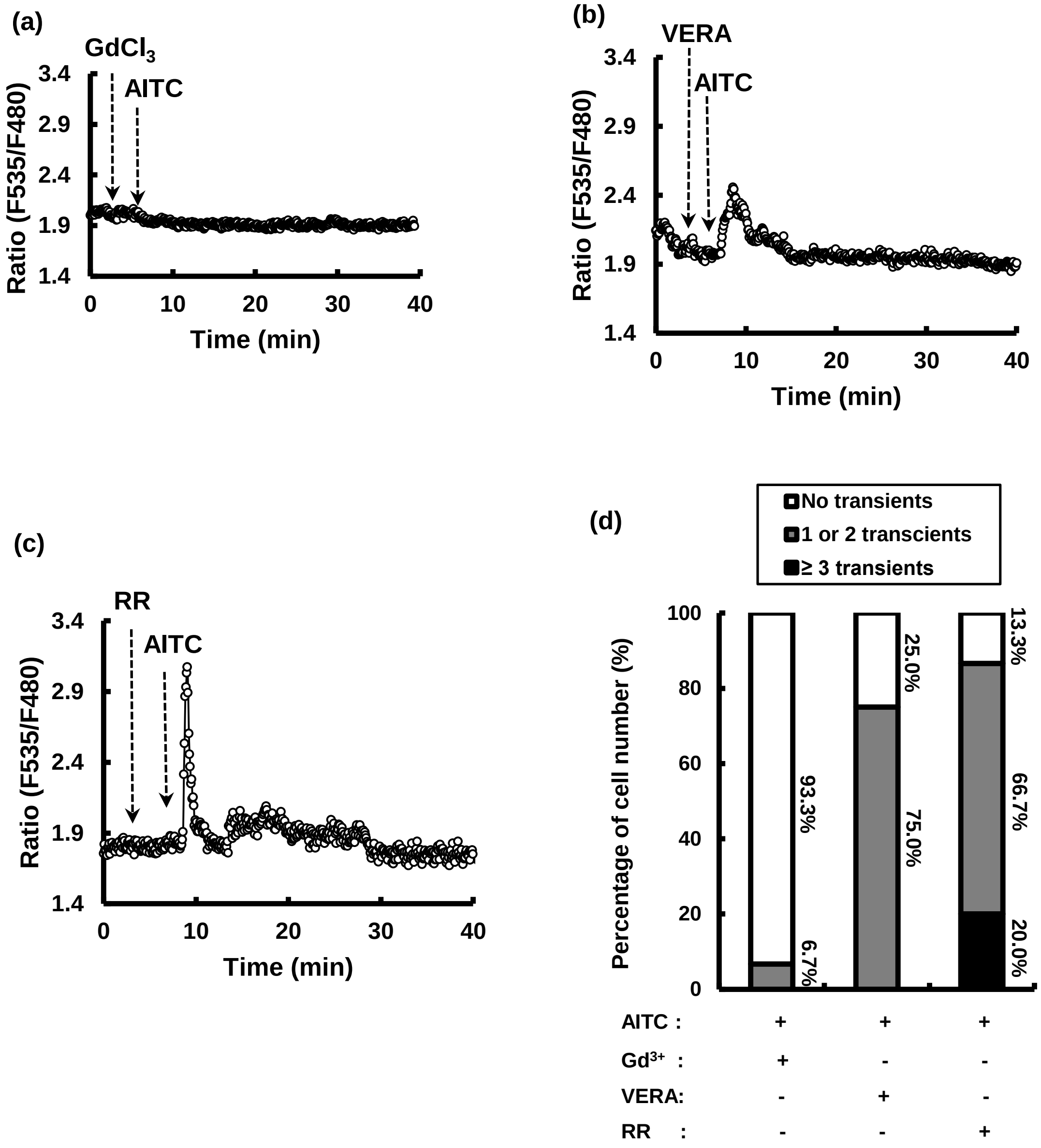

Figure S1. Effects of Gd^{3+}, VERA and RR on AITC-induced elevation of $[Ca^{2+}]_{cyt}$ in guard cells. (a), (b) and (c), representative traces of fluorescence emission ratios (535/480 nm) showing transient $[Ca^{2+}]_{cyt}$ elevations in guard cells. 1 mM Gd^{3+}, 1 mM VERA and 100 µM RR were added 4 min before 50 µM AITC treatment. (d), Percentage of number of guard cells showing different number of transient $[Ca^{2+}]_{cyt}$ elevations in guard cells. $[Ca^{2+}]_{cyt}$ elevations were counted when changes in fluorescence emission ratios were more than or equal to 0.1 U from the baseline.
